# Divisive negative discourse biases social experience: a live experiment at a massive public event

**DOI:** 10.1057/s41599-025-05652-8

**Published:** 2025-08-07

**Authors:** Joaquín Ponferrada, Jeremias Inchauspe, Federico Zimmerman, Gerry Garbulsky, Joaquín Navajas, Adolfo M. García

**Affiliations:** 1 Cognitive Neuroscience Center, University of San Andrés, Buenos Aires, Argentina; 2 Laboratorio de Neurociencia, Escuela de Negocios, Universidad Torcuato di Tella, Ciudad Autónoma de Buenos Aires, Argentina; 3 Harvard Business School, Harvard University, Boston, MA USA; 4 Digital, Data, and Design Institute, Harvard University, Boston, MA USA; 5 Aprender de Grandes, TEDxRíodelaPlata, Ciudad Autónoma de Buenos Aires, Argentina; 6 National Scientific and Technical Research Council, Buenos Aires, Argentina; 7 Departamento de Lingüística y Literatura, Facultad de Humanidades, Universidad de Santiago de Chile, Estación Central, Santiago Chile

**Keywords:** Language and linguistics, Psychology

## Abstract

Linguistic choices, crucially including negatively valenced words and divisive messages, can bias people’s feelings, thoughts, and judgments. However, these phenomena have been typically captured with small groups in controlled settings, casting doubt on their robustness and ecological validity. Here we examined whether such effects hold in a massive public gathering. During a large TEDx event (*n* = 3139), participants engaged in an interactive musical game and then evaluated their perception of (active and vicarious) enjoyment and (ingroup and outgroup) performance through surveys that manipulated (a) the initial framing (‘divisive’ or ‘communal’) and (b) the questions’ valence (‘positive’, ‘neutral’, ‘negative’). Results showed that negatively valenced words reduced enjoyment and performance ratings, particularly under divisive framings. Active enjoyment also decreased under communal framings. These results were corroborated upon adjusting for sociodemographic variables. Briefly, linguistic manipulations of affect immediately altered a crowd’s perception of enjoyment and performance. These insights extend psycholinguistic models and contribute to discussions on public communication.

## Introduction

Language is a powerful modulator of human experience, shaping both individual and shared events (Hoemann and Feldman Barrett 2019; Barrett et al. 2007; Vine et al. 2020). This is especially true of affective language (Satpute and Lindquist 2021). Words evoking emotional and social content influence cardiac (Egger et al. 2019; Weis and Herbert 2017), electrodermal (Egger et al. 2019), and muscular (Egger et al. 2019; Weis and Herbert 2017) activity, impacting ongoing thoughts (Koban et al. 2017; Rohr and Wentura 2022), feelings (Torre and Lieberman 2018), and overall decision-making (Capraro and Vanzo 2019). Yet, such findings come from controlled, low-powered experiments, casting doubt on their robustness in mass gatherings. To tackle this gap, we examined whether affective language influences a crowd’s social experience during a live event.

Knowing whether and how language influences crowds’ attitudes and behaviors is critical to understanding the workings of massive public communication. Rhetorical choices from politicians, for example, can bias the conduct of citizens (Oparinde et al. 2021; Ruggeri et al. 2024), divide them (Adinda Puspa Nur et al. 2022), and make them trust (Loner et al. 2023) or mistrust (Ajzenman et al. 2023) scientific evidence. Such communicative moves often rely on affective language, encompassing emotionally valenced words (like *bad*, *good*, *war*, or *peace*) (Barrett et al. 2007; Barriga-Paulino et al. 2022) and social expressions (terms denoting interpersonal behavior or traits, such as *cooperate*, *fight*, *partner*, or *rival*) (Birba et al. 2023a; Lopes da Cunha et al. 2023b).

In line with the negativity bias (Rozin and Royzman 2001), these units maximally affect behavior when they prompt negative associations (Baumeister et al. 2001). Compared with positive words, negatively valenced items are linked to lower evaluations of active and vicarious enjoyment and/or performance, as seen when questions prime notions of boredom (Hirt et al. 1999), belittle an agent’s status (i.e., *amateur* vs *professional*) (Aydogan et al. 2018; Kroger and Margulis 2017), or emphasize personal shortcomings (Ballard 2019). Also, as shown by affective priming studies, valenced words facilitate responses to same-valence items and interfere with processing of opposite valence items (Spruyt et al. 2002), biasing ensuing judgments toward similarly toned concepts (Janiszewski and Wyer Jr. 2014; Kuperman et al. 2014; Spruyt et al. 2002). Interestingly, however, social judgments and interactive decisions may show similar derogatory effects even in the face of positive words, tentatively reflecting a counter-regulatory system that redirects attention from active to opposite affective states (Rothermund et al. 2008; Schwager and Rothermund 2013).

Language can further impact social experience by foregrounding ingroup or outgroup relations. Although evidence is incipient (Chong and Druckman 2007), judgments of outgroup members are less favorable when elicited under a divisive verbal framing, be it by promoting inter-group competition (Lam and Seaton 2016; Lam and Moodley 2011) or by foregrounding negative stories about rivals (Havard et al. 2021). In addition, outgroup evaluations become lower when elicited through negatively valenced words in descriptions of their members (Schmidtke and Kuperman 2024) or behavior (Hodson et al. 2013). More particularly, specific divisive terms, like “battle”, prompt a rivalry schema and related negative notions, contrasting with words that evoke cooperative frames (Flusberg et al. 2024). Accordingly, framing a given social activity with divisive language (e.g., around the topic of “battle”) or communal language (e.g., focusing on “togetherness”) might influence the very appraisal of the experience.

These effects likely reflect interactive phenomena captured by compatible cognitive models. First, according to the language-as-context framework, linguistic cues supply the conceptual setting that guides emotion construction during comprehension (Barrett et al. 2007). Here, affective words could reactivate consistent conceptual knowledge, shaping emotion perception (Barrett et al. 2007). Second, as proposed by the substance-and-evaluation model, language choices systematically bias judgments about objective events (Leising et al. 2015). Accordingly, words’ evaluative tone interacts with the perceiver’s evaluative attitude to form social judgments (Leising et al. 2015). Importantly, these dynamics have been proposed to operate across large groups (Leising et al. 2015).

Overall, then, negative valence and divisive framings can systematically influence affective experience and performance judgments. However, these effects have been captured in small groups (with means of 19 and 178 participants for valence and framing studies, respectively) (Brooks et al. 2017; McDonald et al. 2021; Balliet et al. 2014), likely yielding underpowered findings. Also, although the meaning of affective words is established through interpersonal exchanges (Borghi 2020), the evidence comes from isolated participants responding to decontextualized stimuli (Brooks et al. 2017; McDonald et al. 2021; Balliet et al. 2014). Thus, little is known about how these effects may manifest in mass gatherings. Moreover, most studies appeal to pre-established and/or longstanding rivalries, based on contrasting political views (Lees and Cikara 2020; Boydstun et al. 2019; Bizer et al. 2011; Rand et al. 2014; Levendusky 2018) or sport team affiliations (Havard et al. 2021; Platow et al. 1999). Consequently, doubts emerge concerning the immediacy of affective language effects.

Here, we examined how negative words and framings impact social experience during a massive public event. The experiment took place during a TEDx event, with 4574 attendees of diverse ages and backgrounds. Unlike most preceding studies, this enabled a novel examination of language effects on crowds’ collective experience while meeting recent calls for testing framing phenomena in real-world (as opposed to highly controlled) settings (Flusberg et al. 2024). Using a “minimal group” paradigm (Tajfel 1970; Brown 2020), we split the audience into two random teams, had them participate in a musical competition, and asked them to complete a survey on active and vicarious enjoyment and performance. The survey featured six versions, including questions with negative, neutral, and positive valence, presented under communal and adversarial framings (emphasizing ingroup affinity and outgroup rivalry, respectively). Based on previous works, we raised two sets of hypotheses.

First, as predicted by the language-as-context framework, enjoyment and performance judgments are prone to negativity bias. We predict that relative to neutral phrasings,

H1. Negatively valenced wordings would reduce perceived enjoyment and performance ratings, both in the active and vicarious conditions; and

H2. Positively valenced wordings would raise these perceptions.

Moreover, we anticipated that, as predicted by the substance-and-evaluation model, divisive framings would:

H3. Strengthen negative-valence effects; and

H4. Attenuate positive-valence effects.

With this framework, we aimed to test whether language-induced affective biases can influence situated mass social experience.

## Methods

**Fig. 1:**
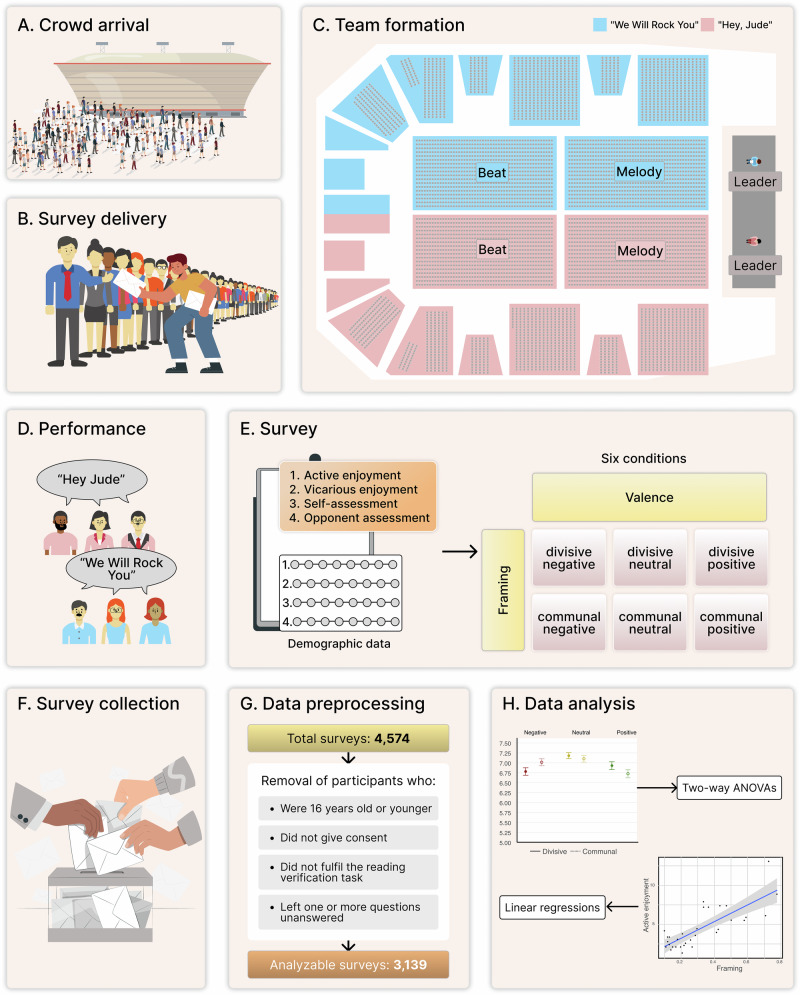
Study design. The experiment began with **A** the crowd's arrival at the rock arena, and **B** the handing of sealed surveys to each attendee. Then, **(C)** participants were divided into two groups to partake in a musical activity requiring them to **D** perform a famous passage from either “Hey, Jude” or “We Will Rock You”. Next, **E** participants completed a survey tapping on active and vicarious enjoyment as well as ingroup and outgroup performance. Different versions of the survey were distributed, varying in their introductory framing (‘divisive’ vs. ‘communal’) and questions’ valence (‘negative’ vs. ‘neutral’ vs. ‘positive’). Finally, surveys were **F** collected, **G** preprocessed, and **H** analyzed via 2×3 ANOVAs and group-wide regressions (adjusting for sociodemographic factors).

As part of a program of live-crowd experiments (Navajas et al. 2019; Navajas et al. 2018; Zimmerman et al. 2022), the study (Fig. 1) took place in November 2022 during a TEDx event at a rock arena in Buenos Aires, Argentina (http://tedxriodelaplata.org/). Preregistration was not feasible since final methodological decisions depended on real-time coordination with the organizers, the very day of the event.

### Participants

Surveys were completed by 4574 participants. To ensure data quality and compliance with inclusion criteria, we discarded participants who (a) were 16 years of age or younger, (b) did not explicitly give their consent, (c) did not fulfill the survey’s reading verification task, or (d) left one or more questions unanswered. This resulted in 3139 valid surveys. A post-hoc power estimation on G*Power (partial η² = 0.01; *f* = 0.10), assuming a deliberately conservative effect (Lakens 2013), yielded a non-centrality parameter λ = 31.71, a critical *F* = 2.22, and a power of 1 − β = 0.997, showing our design is powered to detect even small effects of this magnitude – note that, based on previous TEDx experiments (Navajas et al. 2019; Navajas et al. 2018; Zimmerman et al. 2022), we knew we would have at least 3000 valid participants, rendering a priori power estimations superfluous for our analytical design.

The sample encompassed 1950 women and 1189 men. Their mean age was 33, and their highest education level ranged from primary school (7 years) to postgraduate training (19 years). Based on the survey they received (Table 1), participants were divided into six groups with specific combinations of framing and valence. Such groups did not differ significantly in terms of sex [X^2^(5) = 7.69, *p* = 0.17], age [framing: *F*(1,3133) = 1.83, *p* = 0.18; valence: *F*(2,3133) = 2.03, *p* = 0.13; framing × valence: *F*(2,3133) = 4.69, *p* = 0.01*], or education [framing: *F*(1,3133) = 3.24, *p* = 0.07; valence: *F*(2,3133) = 1.31, *p* = 0.27; framing × valence: *F*(2,3133) = 3.84, *p* = 0.02*] – *p*-values marked with asterisks denote significant effects, followed by non-significant pairwise differences in Tukey’s HSD tests.

**Table 1:** Sociodemographic descriptions by group.

	A	B	C	D	E	F
Framing	Divisive	Divisive	Divisive	Communal	Communal	Communal
Valence	Negative	Neutral	Positive	Negative	Neutral	Positive
Sex (F:M)	304:189	380:231	296:182	357:179	332:207	281:201
Age	32.63 (15.47)	34.76 (15.67)	34.27 (14.83)	33.91 (15.08)	32.35 (15.30)	32.20 (14.85)
Years of education	14.54 (3.20)	14.88 (3.16)	15.05 (3.23)	14.90 (3.09)	14.59 (3.33)	14.71 (3.32)

Education level was established as the highest completed level (primary: 7 years, secondary: 12 years, tertiary: 15 years, university: 17 years, postgraduate: 19 years), transformed into a continuous variable.

*F* Female, *M* Male.

### Materials

Data were collected through an ad-hoc survey with three sections, containing (1) an introductory framing paragraph, (2) four experimental questions, and (3) sociodemographic items. The survey had six versions in a 2 × 3 design, with two framings (‘divisive’, ‘communal’) and three valences (‘positive’, ‘neutral’, ‘negative’). These were randomly distributed in sealed envelopes, alongside a ballpoint pen, when participants entered the event. Participants were instructed not to open or tamper with the envelope in any way until specific instructions were provided on stage.

The surveys’ first section showed a paragraph with a specific framing. Half the surveys had a ‘divisive’ framing (highlighting notions of confrontation) and half had a ‘communal’ framing (highlighting notions of cooperation). As shown in Table 2, this contrast was manifested through opposing words in each paragraph. Participants were instructed to read the text attentively and in full. Compliance was verified by asking the participants to rewrite a given word from the paragraph. Those who left the space blank or wrote in the wrong word were excluded.

**Table 2:** Divisive and communal framings in the surveys.

Divisive framing	Communal framing
You participated in a COMPETITIVE activity. You CONFRONTED a RIVAL who sought to BEAT YOU. You were part of a great musical BATTLE! Think about this experience of CONFRONTATION that you lived and answer the questions with an X.	You participated in a COMMUNAL activity. You INTEGRATED with a GROUP with a SHARED GOAL. You were part of a great musical BAND! Think about this experience of UNION that you lived and answer the questions with an X.

Section 2 consisted of single-sentence questions on active enjoyment, vicarious enjoyment, self-assessment, and opponent assessment. These were manipulated by changing the valence of key words in the questions (Table 3). Each question was followed by a 9-point Likert scale with three labels, two of them placed at the extremes and another at the middle of the scale. For the enjoyment questions, the labels were “Very boring”, “Normal”, and “Very fun”. For the questions regarding performance, the labels were “Very badly”, “Normal”, and “Very well”. In half of the surveys for each valence type, minimum and maximum values for the Likert scales were placed on the left- and right-most ends of the scale, respectively, and reversed in the other half. This was done to prevent any attentional or positional biases in participants’ responses.

**Table 3:** Manipulations of valence in the surveys.

Q	Negative	Neutral	Positive
1	How BORING was it to make music with YOUR GROUP?	How was it to make music with YOUR GROUP?	How FUN was it to make music with YOUR GROUP?
2	How BORING was it to listen to the OTHER GROUP?	How was it to listen to the OTHER GROUP?	How FUN was it to listen to the OTHER GROUP?
3	How BADLY did YOUR GROUP perform?	How did YOUR GROUP perform?	How WELL did YOUR GROUP perform?
4	How BADLY did the OTHER GROUP perform?	How did the OTHER GROUP perform?	How WELL did the OTHER GROUP perform?

The last section tapped into the demographic profile. Age was queried through an open field requiring a single value. Sex was captured via a multiple-choice item (“Male”, “Female”, “Other”, “I’d rather not say”). Education level was also queried through a list of options (Primary, Secondary, Tertiary, University, Postgraduate).

### Procedure

Two presenters walked on stage and told the crowd that they would participate in a musical activity. The crowd was divided into two groups of about 2287 people each, based on their position on either side of the central aisle. They were informed that the loudest and most energetic group would win and enter a raffle for TEDx merchandise. Then, the leader of each group joined the stage and stood on opposite ends, facing their respective group. To further promote distinct group affiliations, each leader carried a specific logo on their T-shirts, which was also displayed on large on-stage screens. Each group greeted its leader enthusiastically.

To strengthen ingroup affiliation, each leader asked their group to scream together as loudly as they could. Then, one group was assigned the chorus to The Beatles’ “Hey, Jude”, while the other was given the chorus of Queen’s “We Will Rock You”. Each group was also divided into percussion and melody: the people in the top seats were asked to play a beat with their hands on their chests, while those in the lower seats had to sing the melody.

The leaders briefly demonstrated how to perform the percussion and melody parts of their song. Participants practiced their parts and were asked to stand up for the performance. The “Hey, Jude” group performed first, and the “We Will Rock You” group went second. Each group sang for roughly 90 s, loudly and enthusiastically. The activity finished with a round of applause from the whole crowd as the leaders left the stage.

The presenters then asked participants to grab the envelopes and wait for their signal to open them. They indicated that participants had to (1) read each instruction carefully, (2) abstain from copying responses from another participant, and (3) refrain from asking for help. They were also informed that participation was completely voluntary and that they could opt out at any moment. They were then asked to open the envelopes and given 4 min to complete the questionnaire. Once the time was up, they were instructed to put the survey back in the envelope and to pass it toward the right end of their row, for collection by our team members.

### Data analysis

Each question was analyzed via a two-way analysis of variance (ANOVA), with the factors framing (‘divisive’, ‘communal’) and valence (‘positive’, ‘neutral’, ‘negative’). This allowed us to examine the interplay of both variables in a between-subjects design. To this end, participants were divided into six sociodemographically matched groups (divisive-negative, divisive-neutral, divisive-positive, communal-negative, communal-neutral, communal-positive) –see “Participants” section. No outliers were detected at a threshold of 3 standard deviations from each group’s mean. Interaction effects were examined via Tukey’s HSD post-hoc tests. Alpha levels were set at *p* < .05. Effect sizes were calculated through partial eta squared (η^2^) tests for ANOVA results and Cohen’s *d* for pairwise comparisons.

To further the robustness of findings, we also examined the influence of framing (‘divisive’, ‘communal’) and valence (‘negative’, ‘neutral’, ‘positive’) across the entire sample via separate linear regression models for each question (‘active enjoyment’, ‘vicarious enjoyment’, ‘self-assessment’, ‘opponent assessment’). Variables of interest were included as fixed effects. Dummy variables were created for the negative and positive valences, while the neutral one was set as the reference category. Also, we created a dummy variable for the ‘communal’ framing, with the ‘divisive’ framing established as the reference point for comparison. Additionally, the factors ‘age’, ‘sex’, and ‘years of education’ were included as control variables to test for robustness against sociodemographic confounds. We applied a Bonferroni correction to the alpha level, setting it at 0.017 to mitigate the risk of Type-I errors while conducting multiple comparisons. This adjustment ensures a more stringent threshold for statistical significance (Armstrong 2014; Cabin and Mitchell 2000). All analyses were run on RStudio 2022.12.0 (R Core Team 2022).

## Results

### Between-group comparisons

**Fig. 2:**
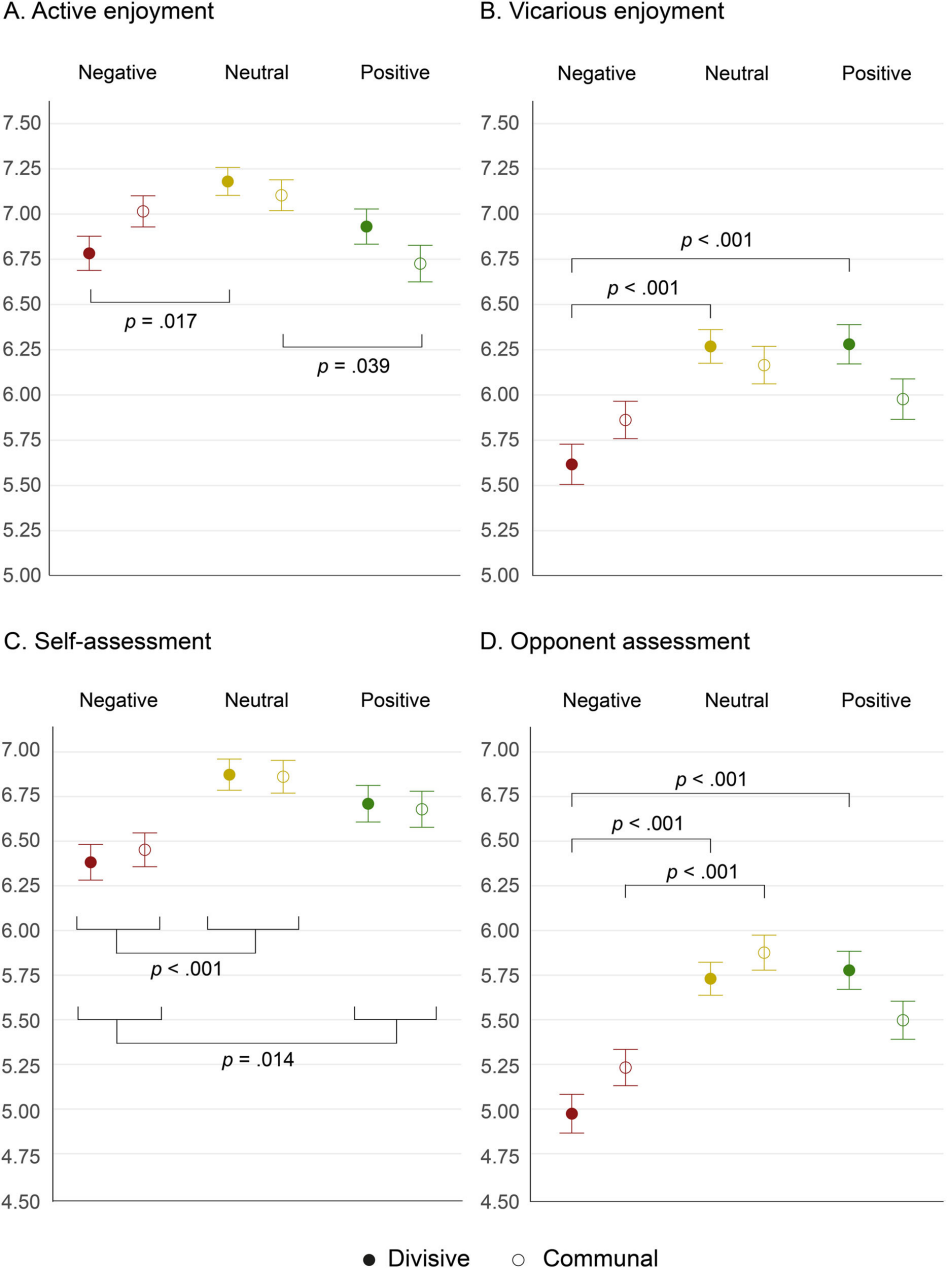
ANOVA results. Significant group-level results based on two-way ANOVAs, considering framing (divisive, communal) and word valence (negative, neutral, positive). Participants reported **A** lower active enjoyment when faced with valenced wordings, **B** lower vicarious enjoyment when faced with negative words under a divisive framing, **C** lower ratings of their own group’s performance when faced with negative words, irrespective of framing, and **D** lower ratings of the opposing group’s performance when faced with negative words under the divisive framing. Filled markers indicate divisive framing, while open markers indicate communal framing. Brackets indicate significant pairwise differences in interaction effects.

Regarding active enjoyment (Fig. 2A), we observed a non-significant effect of framing [*F*(1,3133) = 3.292, *p* = 0.070, η^2^ = 0.000], a significant effect of valence [*F*(2,3133) = 4.662, *p* = 0.010, η^2^ = 0.004], and a significant interaction between both variables [*F*(2,3133) = 3.024, *p* = 0.049, η^2^ = 0.002]. A post-hoc analysis (Tukey’s HSD: MSE = 29.24, *df* = 2) revealed significantly lower enjoyment scores for negative than neutral questions (*p* = 0.018, *d* = 0.121) and for positive than neutral questions (*p* = 0.001, *d* = 0.154). Pairwise comparisons (Tukey’s HSD: MSE = 12.69, *df* = 2) showed significantly lower enjoyment scores for the divisive-negative than for the divisive-neutral condition (*p* = 0.017, *d* = 0.199) and significantly higher enjoyment scores for the communal-neutral than for the communal-positive condition (*p* = 0.039, *d* = 0.180). Every other pairwise comparison revealed non-significant differences (all *p-*values > 0.120).

Considering vicarious enjoyment (Fig. 2B), we found non-significant effects of framing [*F*(1,3133) = 2.689, *p* = 0.101, η^2^ = 0.000] and valence [*F*(2,3133) = 2.189, *p* = 0.112, η^2^ = 0.007], alongside a significant interaction between both factors [*F*(2,3133) = 3.358, *p* = 0.035, η^2^ = 0.002]. Pairwise comparisons (Tukey’s HSD: MSE = 19.29, *df* = 2) revealed significantly lower enjoyment scores for the divisive-negative than for the divisive-neutral (*p* < 0.001, *d* = 0.274) and the divisive-positive (*p* < 0.001, *d* = 0.274) conditions. Every other pairwise comparison revealed uninterpretable or non-significant differences (all *p-*values > 0.062).

On self-assessment (Fig. 2C), we found a non-significant effect of framing [*F*(1,3133) = 0.260, *p* = 0.610, η^2^ = 0.000] and a significant effect of valence [*F*(2,3133) = 4.701, *p* = 0.009, η^2^ = 0.007]. The interaction effect between framing and valence was non-significant [*F*(2,3133) = 0.151, *p* = 0.860, η^2^ = 0.000]. Post-hoc analyses (Tukey’s HSD: MSE = 53.76, *df* = 2) revealed significantly lower performance evaluations for the negative condition than for the neutral (*p* < 0.001, *d* = 0.206) and positive (*p* = 0.014, *d* = 0.124) conditions.

When analyzing opponent assessment (Fig. 2D), we found a non-significant effect of framing [*F*(1,3133) = 3.151, *p* = 0.076, η^2^ = 0.000], a significant effect of valence [*F*(2,3133) = 10.336, *p* < 0.001, η^2^ = 0.016], and a significant interaction between both factors [*F*(2,3133) = 3.668, *p* = 0.026, η^2^ = 0.002]. Post-hoc analyses (Tukey’s HSD: MSE = 135.23, *df* = 2) showed significantly lower scores for the negative valence than for the neutral (*p* < 0.001, *d* = 0.296) and positive (*p* < 0.001, *d* = 0.223) valences. Pairwise comparisons (Tukey’s HSD: MSE = 19.42, *df* = 2) revealed lower scores in the communal-negative than in the communal-neutral condition (*p* < 0.001, *d* = 0.278), in the divisive-negative than in the divisive-neutral condition (*p* < 0.001, *d* = 0.323), and in the divisive-negative than in the divisive-positive condition (*p* < 0.001, *d* = 0.338). Every other pairwise comparison revealed non-significant differences (all *p-*values > 0.100). Results remained largely similar upon covarying for demographic factors (Supplementary Tables S1–S4).

### Whole-group regressions

Linear regressions (Table 4) revealed no significant differences between the ‘divisive’ and the ‘communal’ framing on all four variables, namely: active enjoyment [*β* = −0.01, SE = 0.07, *t*(3131) = −0.2, *p* = 0.86], vicarious enjoyment [*β* = −0.01, SE = 0.08, *t*(3132) = −0.1, *p* = 0.89], self-assessment [*β* = 0.01, SE = 0.08, *t*(3132) = 0.1, *p* = 0.89], opponent assessment [*β* = 0.09, SE = 0.08, *t*(3132) = 1.2, *p* = 0.25]. Conversely, questions asked with negative valence had a significant negative effect on active enjoyment [*β* = −0.25, SE = 0.09, *t*(3131) = −2.9, *p* = 0.004], vicarious enjoyment [*β* = −0.48, SE = 0.10, *t*(3132) = −4.9, *p* < 0.0001], self-assessment [*β* = −0.45, SE = 0.09, *t*(3132) = −4.9, *p* < 0.001], and opponent assessment [*β* = −0.68, SE = 0.09, *t*(3132) = −7.2, *p* < .001]. Questions asked with positive valence had a significant negative effect on active enjoyment [*β* = −0.29, SE = 0.09, *t*(3131) = −3.3, *p* = 0.001] and non-significant effects on any other variable (all *p*-values > 0.113). All these results were adjusted for ‘age’, ‘sex’, and ‘years of education’.

**Table 4:** Regression results.

Term	estimate	*SD*	*t*	*p*
**Active enjoyment**				
(Intercept)	7.966	0.186	42.85	<0.001
Framing	−0.013	0.072	−0.17	0.862
Negative valence	−0.251	0.087	−2.89	0.004
Positive valence	−0.294	0.089	−3.31	0.001
Sex	−0.574	0.075	−7.66	<0.001
Age	0.009	0.003	2.94	0.003
Years of education	−0.060	0.014	−4.33	<0.001
**Vicarious enjoyment**				
(Intercept)	4.192	0.210	19.99	<0.001
Framing	−0.012	0.082	−0.14	0.888
Negative valence	−0.478	0.098	−4.86	<0.001
Positive valence	−0.081	0.100	−0.81	0.421
Sex	−0.591	0.085	−6.99	<0.001
Age	0.030	0.003	9.02	<0.001
Years of education	0.085	0.016	5.46	<0.001
**Self-assessment**				
(Intercept)	7.782	0.196	39.62	<0.001
Framing	0.010	0.077	0.13	0.893
Negative valence	−0.454	0.092	−4.93	<0.001
Positive valence	−0.148	0.094	−1.58	0.113
Sex	−0.537	0.079	−6.78	<0.001
Age	0.009	0.003	2.77	0.006
Years of education	−0.069	0.015	−4.70	<0.001
**Opponent assessment**				
(Intercept)	3.950	0.202	19.53	<0.001
Framing	0.091	0.079	1.15	0.248
Negative valence	−0.684	0.095	−7.22	<0.001
Positive valence	−0.150	0.097	−1.56	0.120
Sex	−0.433	0.081	−5.31	<0.001
Age	0.032	0.003	10.08	<0.001
Years of education	0.061	0.015	4.02	<0.001

## Discussion

We examined the impact of affective language on mass social experience. Negative words reduced enjoyment and performance judgments, especially under a divisive framing. The same effect emerged for positive words under a communal framing. Results held upon adjusting for demographic variables. Below, we discuss our findings.

Judgments of both active and vicarious enjoyment were reduced by negative words. Compatibly, overall enjoyment has been shown to decrease in the face of negatively framed instructions (Hirt et al. 1999) or non-flattering descriptions (Aydogan et al. 2018; Kroger and Margulis 2017). Such findings arguably reflect the well-reported negativity bias, whereby perceptions and behaviors are more influenced by negative than by positive or neutral information (Rozin and Royzman 2001; Baumeister et al. 2001). Crucially, we showed that these phenomena are not confined to controlled laboratory conditions, as they emerged in a large crowd setting, attesting to their generalizability and real-world relevance.

The effects above emerged only under the divisive framing, which emphasized between-group rivalry rather than within-group integration (Lam and Seaton 2016). Contextual manipulations during emotion word processing can alter attention (Lee and Potter 2020), memory recall (Lee and Potter 2020), valence perceptions (Zhang et al. 2021), and semantic associations (Hoemann and Feldman Barrett 2019). In particular, the “battle” semantic frame, used in our study, has been shown to direct attention to congruent cues of rivalry, heightening negative affect (Flusberg et al. 2024). Indeed, words’ perceived negativity increases when prior instructions emphasize negativity (Briesemeister et al. 2012). Such processes may be partly driven by affective priming. Indeed, negatively valenced words can pre-activate similarly valenced concepts, easing their retrieval (Kuperman et al. 2014; Spruyt et al. 2002). In our experiment, negative wordings may have prompted affectively congruent notions (e.g., boredom, ineffectiveness) over incongruent ones (e.g., fun, effectiveness), biasing participants’ judgments toward lower enjoyment and performance ratings.

Together, these findings can be interpreted through the Substance-and-Evaluation Model, which posits that individual judgments are influenced by actual experience (substance) and evaluative attitude, which interact with word valence (evaluative tone) (Leising et al. 2015). Here, competitive evaluative attitudes induced by the divisive framing would converge with words’ negative evaluative tone, augmenting negative schemas and reducing perceptions of enjoyment (Leising et al. 2015). Overall, our findings corroborate the context-dependency of valence effects, showing that such integration operates even at the crowd level.

Negative words also reduced self-assessment and opponent assessments, but these effects were uninfluenced by framing. Contextual interpersonal information, then, would impact hedonic experience more than it does performance judgments. In terms of the Substance-and-Evaluation Model, performance ratings would be influenced by words’ evaluative tone irrespective of evaluative attitude (Leising et al. 2015). Indeed, negative valence reduces performance ratings when participants are primed with negative information about a performer’s status or skill (Aydogan et al. 2018; Kroger and Margulis 2017) or when drawbacks are emphasized (Ballard 2019). Thus, as seen in experimental settings, the interaction of framing and word valence in mass gatherings would partly depend on the domain being judged.

The only exception to this systematic negativity bias concerned active enjoyment judgments, which were also decreased under the communal framing. Though seemingly paradoxical, such a result is consistent with the idea of a counter-regulatory system, where framing has an incongruent effect on affective processing in cases of motivational outcome focus (Rothermund et al. 2008; Schwager and Rothermund 2013). Rather than excluding valence-incongruent details, counter-regulation would supplement priming-driven congruency biases, leading social judgments to be jointly shaped by matching and opposing affective cues (Schwager and Rothermund 2013). Under such conditions, this view would have it, stimuli would shift attention away from the communal experience, resulting in a lower perception of enjoyment. Still, such interpretation remains conjectural and should be directly tackled in future research through strategic designs.

The key findings above were replicated in our regression analyses, run over the entire dataset (*n* = 3139) while adjusting for sociodemographic variables. This is a non-trivial finding, since emotional language effects in certain tasks or settings can be modulated by sex (Lin et al. 2021; Teismann et al. 2020), age (Kyröläinen et al. 2021; Teismann et al. 2020), and education (Lane et al. 1998; Nandrino et al. 2013). The consistency of our findings despite variability in these factors speaks to the potential generalizability of situated affective language effects.

Such effects, it must be noted, had small sizes. This is consistent with the framing literature (Flusberg et al. 2024) and with studies on other forms of mass affective behavior (Kramer et al. 2014). As proposed in such research, small effect sizes prove informative in crowd-level studies, as affective dependent variables are difficult to influence due to the range of factors modulating mood (Golder and Macy 2011). Observing these effects under such minimal manipulations as single words further attests to the aggregate collective consequences of language choices (Bond et al. 2012; Prentice and Miller 1992).

The observed results can be comprehensively understood through the language-as-context framework (Barrett et al. 2007), which posits that affective language constitutes a frame of reference shaping our emotional experience (Fugate et al. 2018; Lindquist and Gendron 2013; Lindquist et al. 2015). Language would act as a top-down constraint on emotion perception by providing conceptual categories that help interpret sensory information (Barrett et al. 2011; Fugate et al. 2018). In particular, valenced words, rooted in shared social experience (Borghi 2020), can swiftly redirect attention toward their core semantic associations (Lupyan 2016) and activate relevant interpretive schemas (Dove 2023). Here, we surmise, negative wordings and divisive framings would anchor ongoing experience in schemas typically associated with unpleasant feelings and suboptimal outcomes, biasing the perception, recollection, and/or rating of enjoyment and performance under both ingroup and outgroup perspectives. This interpretation invites further research on the real-world implications of discourse-level contextual manipulations.

More generally, our results carry three main implications. First, they stemmed from a large sample size, attesting to their robustness and tackling a core challenge in cognitive science (Button et al. 2013; Kousta 2017; Nosek et al. 2022). Second, they emerged during a live event. While most affective language research occurs in controlled conditions and often fails to replicate elsewhere (Flusberg et al. 2024), our findings emerged in a real-world gathering (Navajas et al. 2019; Navajas et al. 2018; Zimmerman et al. 2022), meeting the recent call for well-powered framing studies “in the wild” (Flusberg et al. 2024). More particularly, our study shows language-induced biases can immediately modulate crowds’ collective experiences based on arbitrary ingroup/outgroup divides. Extending findings on isolated participants, this suggests that affective language choices can modulate mass behavior by prompting capricious antagonism and negative affect. Lastly, unlike works targeting interpersonal dynamics via deep-rooted (e.g., ideological, political) rivalries (Bizer et al. 2011; Boydstun et al. 2019; Havard et al. 2021; Lees and Cikara 2020; Levendusky 2018; Platow et al. 1999; Rand et al. 2014), ours shows that interpersonal framings can bias enjoyment judgments without foregrounding longstanding cultural affiliations or antagonisms. This suggests that oppositional affective biases could be created on the fly, reinforcing the strong impact of language on mass social dynamics. Beyond classical findings, then, affective language effects seem typified by their generalizability, representativeness, and immediacy, inviting extensions of relevant models in the field.

## Limitations and further research

Our study is not without limitations. First, due to time constraints during the live event, our survey was restricted to only a few questions. Future research should add further items capturing a broader range of individual and vicarious experiences, ideally incorporating responses beyond Likert scales. Second, the massive event’s conditions may have impacted participants’ attention and deliberation. Though this is part of the tradeoff of conducting ecological research, further work could mitigate these issues through logistical strategies (e.g., pooling subgroups together in small rooms for task completion). Third, we were unable to measure relevant psychological aspects, such as baseline emotional states (Watson et al. 1988), socio-cognitive profiles (Baron-Cohen et al. 2001), or personality traits (Gosling et al. 2003). Such measures could yield deeper insights into inter-individual variability during affective language processing. Finally, we lacked non-verbal manipulations (e.g., affective images or sounds), inviting new research on whether similar framing and valence effects emerge beyond the linguistic domain.

## Conclusion

Affective language manipulations seem to modulate mass social experience. During a multitudinous live event, negatively valenced words reduced enjoyment (under adversarial framings) and performance judgments (across interpersonal framings), irrespective of demographics. These findings open a promising agenda to study the real-life implications of verbal manipulations in public settings.

## Supplementary information

Supplementary information

## Data Availability

All experimental data, questionnaires, and analysis scripts are fully available online at: https://osf.io/f8ytx.
