# Supplementary material for: Divisive negative discourse biases social experience: a live experiment at a massive public event
Source: Humanit Soc Sci Commun. 2025 Aug 7;12(1):1273. doi: 10.1057/s41599-025-05652-8 (PMC12331517; doi:10.1057/s41599-025-05652-8)
Supplement: Supplementary file 1 — Supplementary information [file 41599_2025_5652_MOESM1_ESM.pdf]

Supplementary Information

Supplementary Table S1. Q1: Full ANCOVA results.

	F	Pr(>F)	ηp²	Pairwise comparisons		*t*	*p*	Cohen's *d*
**Covariation with sex**								
Framing	0.058	0.810	< 0.001	Com-Neg	Div-Neg	1.620	1.000	0.101
Valence	6.972	< 0.001	0.004	Com-Neg	Com-Neu	-0.934	1.000	-0.057
Framing:Valence	2.466	0.085	0.002	Com-Neg	Com-Pos	1.911	0.842	0.120
				Div-Neg	Com-Neg	-2.538	0.168	-0.158
				Div-Neg	Div-Neu	-3.205	0.020	-0.194
				Div-Neg	Div-Pos	-1.124	1.000	-0.072
				Com-Neu	Div-Neu	-0.607	1.000	-0.036
				Com-Neu	Com-Pos	2.825	0.071	0.177
				Div-Neu	Div-Pos	1.996	0.690	0.122
				Com-Pos	Div-Pos	-1.411	1.000	-0.091
**Covariation with age**								
Framing	0.040	0.841	< 0.001	Com-Neg	Div-Neg	1.799	1.000	0.112
Valence	6.915	0.001	0.004	Com-Neg	Com-Neu	-0.729	1.000	-0.045
Framing:Valence	2.940	0.053	0.002	Com-Neg	Com-Pos	2.225	0.393	0.140
				Div-Neg	Com-Neg	-2.517	0.178	-0.157
				Div-Neg	Div-Neu	-3.174	0.023	-0.192
				Div-Neg	Div-Pos	-1.107	1.000	-0.071
				Com-Neu	Div-Neu	-0.599	1.000	-0.035
				Com-Neu	Com-Pos	2.940	0.050	0.184
				Div-Neu	Div-Pos	1.985	0.708	0.121
				Com-Pos	Div-Pos	-1.525	1.000	-0.099
**Covariation with years of education**								
Framing	0.069	0.793	< 0.001	Com-Neg	Div-Neg	1.900	0.863	0.119
Valence	6.821	0.001	0.004	Com-Neg	Com-Neu	-0.637	1.000	-0.039
Framing:Valence	3.347	0.035	0.002	Com-Neg	Com-Pos	2.292	0.330	0.144
				Div-Neg	Com-Neg	-2.528	0.173	-0.158
				Div-Neg	Div-Neu	-3.288	0.015	-0.199
				Div-Neg	Div-Pos	-1.245	1.000	-0.080
				Com-Neu	Div-Neu	-0.704	1.000	-0.042
				Com-Neu	Com-Pos	2.916	0.054	0.183
				Div-Neu	Div-Pos	1.952	0.766	0.119
				Com-Pos	Div-Pos	-1.630	1.000	-0.105

All *p*-values are reported following Bonferroni correction. Com: Communal; Div: divisive; Neg: negative; Neu: neutral; Pos: positive.

Supplementary Table S2. Q2: Full ANCOVA results.

	F	Pr(>F)	ηp²	Pairwise comparisons		*t*	*p*	Cohen's *d*
**Covariation with sex**								
Framing	0.422	0.516	< 0.001	Com-Neg	Div-Neg	1.434	1.000	0.090
Valence	13.058	< 0.001	0.008	Com-Neg	Com-Neu	-2.319	0.307	-0.142
Framing:Valence	2.744	0.064	0.002	Com-Neg	Com-Pos	-1.139	1.000	-0.072
				Div-Neg	Com-Neg	-3.707	0.003	-0.231
				Div-Neg	Div-Neu	-4.507	< 0.001	-0.273
				Div-Neg	Div-Pos	-4.340	< 0.001	-0.279
				Com-Neu	Div-Neu	-0.708	1.000	-0.042
				Com-Neu	Com-Pos	1.115	1.000	0.070
				Div-Neu	Div-Pos	-0.094	1.000	-0.006
				Com-Pos	Div-Pos	-1.820	1.000	-0.118
**Covariation with age**								
Framing	0.016	0.898	< 0.001	Com-Neg	Div-Neg	1.336	1.000	0.083
Valence	12.262	< 0.001	0.008	Com-Neg	Com-Neu	-2.593	0.144	-0.158
Framing:Valence	1.963	0.141	0.001	Com-Neg	Com-Pos	-1.269	1.000	-0.080
				Div-Neg	Com-Neg	-3.877	0.002	-0.242
				Div-Neg	Div-Neu	-4.028	< 0.001	-0.244
				Div-Neg	Div-Pos	-4.014	< 0.001	-0.258
				Com-Neu	Div-Neu	-0.041	1.000	-0.002
				Com-Neu	Com-Pos	1.253	1.000	0.079
				Div-Neu	Div-Pos	-0.225	1.000	-0.014
				Com-Pos	Div-Pos	-1.466	1.000	-0.095
**Covariation with years of education**								
Framing	0.205	0.651	< 0.001	Com-Neg	Div-Neg	1.254	1.000	0.078
Valence	12.011	< 0.001	0.008	Com-Neg	Com-Neu	-2.515	0.179	-0.153
Framing:Valence	2.083	0.125	0.001	Com-Neg	Com-Pos	-1.017	1.000	-0.064
				Div-Neg	Com-Neg	-3.719	0.003	-0.232
				Div-Neg	Div-Neu	-4.197	< 0.001	-0.254
				Div-Neg	Div-Pos	-3.839	0.002	-0.247
				Com-Neu	Div-Neu	-0.379	1.000	-0.022
				Com-Neu	Com-Pos	1.430	1.000	0.090
				Div-Neu	Div-Pos	0.124	1.000	0.008
				Com-Pos	Div-Pos	-1.619	1.000	-0.105

All *p*-values are reported following Bonferroni correction. Com: Communal; Div: divisive; Neg: negative; Neu: neutral; Pos: positive.

Supplementary Table S3. Q3: Full ANCOVA results.

	F	Pr(>F)	η_p^2	Pairwise comparisons		*t*	*p*	Cohen's *d*
**Covariation with sex**								
Framing	0.010	0.920	< 0.001	Com-Neg	Div-Neg	0.326	1.000	0.020
Valence	12.353	< 0.001	0.008	Com-Neg	Com-Neu	-3.272	0.016	-0.200
Framing:Valence	0.055	0.947	< 0.001	Com-Neg	Com-Pos	-1.970	0.735	-0.124
				Div-Neg	Com-Neg	-3.531	0.006	-0.220
				Div-Neg	Div-Neu	-3.699	0.003	-0.224
				Div-Neg	Div-Pos	-2.335	0.294	-0.150
				Com-Neu	Div-Neu	-0.066	1.000	-0.004
				Com-Neu	Com-Pos	1.211	1.000	0.076
				Div-Neu	Div-Pos	1.213	1.000	0.074
				Com-Pos	Div-Pos	-0.089	1.000	-0.006
**Covariation with age**								
Framing	0.015	0.903	< 0.001	Com-Neg	Div-Neg	0.504	1.000	0.031
Valence	11.507	< 0.001	0.007	Com-Neg	Com-Neu	-3.065	0.033	-0.187
Framing:Valence	0.145	0.865	< 0.001	Com-Neg	Com-Pos	-1.656	1.000	-0.104
				Div-Neg	Com-Neg	-3.507	0.007	-0.219
				Div-Neg	Div-Neu	-3.684	0.004	-0.223
				Div-Neg	Div-Pos	-2.322	0.304	-0.149
				Com-Neu	Div-Neu	-0.079	1.000	-0.005
				Com-Neu	Com-Pos	1.325	1.000	0.083
				Div-Neu	Div-Pos	1.213	1.000	0.074
				Com-Pos	Div-Pos	-0.212	1.000	-0.014
**Covariation with years of education**								
Framing	0.005	0.945	< 0.001	Com-Neg	Div-Neg	0.615	1.000	0.038
Valence	11.615	< 0.001	0.007	Com-Neg	Com-Neu	-2.971	0.045	-0.181
Framing:Valence	0.252	0.777	< 0.001	Com-Neg	Com-Pos	-1.597	1.000	-0.100
				Div-Neg	Com-Neg	-3.525	0.006	-0.220
				Div-Neg	Div-Neu	-3.802	0.002	-0.230
				Div-Neg	Div-Pos	-2.475	0.201	-0.159
				Com-Neu	Div-Neu	-0.179	1.000	-0.011
				Com-Neu	Com-Pos	1.293	1.000	0.081
				Div-Neu	Div-Pos	1.167	1.000	0.071
				Com-Pos	Div-Pos	-0.316	1.000	-0.020

All *p*-values are reported following Bonferroni correction. Com: Communal; Div: divisive; Neg: negative; Neu: neutral; Pos: positive.

Supplementary Table S4. Q4: Full ANCOVA results.

	F	Pr(>F)	ηp^2	Pairwise comparisons		*t*	*p*	Cohen's *d*
**Covariation with sex**								
Framing	0.235	0.628	< 0.001	Com-Neg	Div-Neg	1.614	1.000	0.101
Valence	27.396	< 0.001	0.017	Com-Neg	Com-Neu	-4.722	< 0.001	-0.288
Framing:Valence	3.231	0.040	0.002	Com-Neg	Com-Pos	-2.101	0.536	-0.132
				Div-Neg	Com-Neg	-6.241	< 0.001	-0.389
				Div-Neg	Div-Neu	-5.363	< 0.001	-0.325
				Div-Neg	Div-Pos	-5.384	< 0.001	-0.346
				Com-Neu	Div-Neu	1.087	1.000	0.064
				Com-Neu	Com-Pos	2.490	0.192	0.156
				Div-Neu	Div-Pos	-0.343	1.000	-0.021
				Com-Pos	Div-Pos	-1.748	1.000	-0.113
**Covariation with age**								
Framing	1.093	0.296	< 0.001	Com-Neg	Div-Neg	1.470	1.000	0.092
Valence	27.290	< 0.001	0.017	Com-Neg	Com-Neu	-5.146	< 0.001	-0.314
Framing:Valence	2.950	0.053	0.002	Com-Neg	Com-Pos	-2.363	0.273	-0.148
				Div-Neg	Com-Neg	-6.511	< 0.001	-0.406
				Div-Neg	Div-Neu	-4.913	< 0.001	-0.298
				Div-Neg	Div-Pos	-5.100	< 0.001	-0.328
				Com-Neu	Div-Neu	1.827	1.000	0.108
				Com-Neu	Com-Pos	2.642	0.124	0.166
				Div-Neu	Div-Pos	-0.488	1.000	-0.030
				Com-Pos	Div-Pos	-1.352	1.000	-0.087
**Covariation with years of education**								
Framing	0.467	0.494	< 0.001	Com-Neg	Div-Neg	1.421	1.000	0.089
Valence	26.686	< 0.001	0.017	Com-Neg	Com-Neu	-4.982	< 0.001	-0.304
Framing:Valence	2.872	0.057	0.002	Com-Neg	Com-Pos	-2.063	0.588	-0.130
				Div-Neg	Com-Neg	-6.302	< 0.001	-0.393
				Div-Neg	Div-Neu	-5.094	< 0.001	-0.309
				Div-Neg	Div-Pos	-4.944	< 0.001	-0.318
				Com-Neu	Div-Neu	1.424	1.000	0.084
				Com-Neu	Com-Pos	2.784	0.081	0.175
				Div-Neu	Div-Pos	-0.150	1.000	-0.009
				Com-Pos	Div-Pos	-1.540	1.000	-0.099

All *p*-values are reported following Bonferroni correction. Com: Communal; Div: divisive; Neg: negative; Neu: neutral; Pos: positive.
